# Globus Pallidus Internus Deep Brain Stimulation for Dystonic Opisthotonus in Adult-Onset Dystonia: A Personalized Approach

**DOI:** 10.3389/fnhum.2021.683545

**Published:** 2021-06-10

**Authors:** Kantharuby Tambirajoo, Luciano Furlanetti, Michael Samuel, Keyoumars Ashkan

**Affiliations:** ^1^Department of Neurosurgery, King’s College Hospital, London, United Kingdom; ^2^Department of Basic and Clinical Neuroscience, IoPPN, King’s College London, London, United Kingdom; ^3^Department of Neurology, King’s College Hospital, London, United Kingdom

**Keywords:** axial dystonia, movement disorders, globus pallidus internus, deep brain stimulation, opisthotonus

## Abstract

**Introduction:**

Dystonic opisthotonus is defined as a backward arching of the neck and trunk, which ranges in severity from mild backward jerks to life-threatening prolonged severe muscular spasms. It can be associated with generalized dystonic syndromes or, rarely, present as a form of axial truncal dystonia. The etiologies vary from idiopathic, genetic, tardive, hereditary-degenerative, or associated with parkinsonism. We report clinical cases of dystonic opisthotonus associated with adult-onset dystonic syndromes, that benefitted from globus pallidus internus (GPi) deep brain stimulation (DBS).

**Methods:**

Clinical data from patients with dystonic syndromes who underwent comprehensive medical review, multidisciplinary assessment, and tailored medical and neurosurgical managements were prospectively analyzed. Quantification of dystonia severity pre- and postoperatively was performed using the Burke-Fahn-Marsden Dystonia Rating Scale and quantification of overall pain severity was performed using the Visual Analog Scale.

**Results:**

Three male patients, with age of onset of the dystonic symptoms ranging from 32 to 51 years old, were included. Tardive dystonia, adult-onset dystonia-parkinsonism and adult-onset idiopathic axial dystonia were the etiologies identified. Clinical investigation and management were tailored according to the complexity of the individual presentations. Although they shared common clinical features of adult-onset dystonia, disabling dystonic opisthotonus, refractory to medical management, was the main indication for GPi-DBS in all patients presented. The severity of axial dystonia ranged from disturbance of daily function to life-threatening truncal distortion. All three patients underwent bilateral GPi DBS at a mean age of 52 years (range 48–55 years), after mean duration of symptoms prior to DBS of 10.7 years (range 4–16 years). All patients showed a rapid and sustained clinical improvement of their symptoms, notably of the dystonic opisthotonos, at postoperative follow-up ranging from 20 to 175 months. In some, the ability to resume activities of daily living and reintegration into the society was remarkable.

**Conclusion:**

Adult-onset dystonic syndromes predominantly presenting with dystonic opisthotonus are relatively rare. The specific nature of dystonic opisthotonus remains a treatment challenge, and thorough investigation of this highly disabling condition with varying etiologies is often necessary. Although patients may be refractory to medical management and botulinum toxin injection, Globus pallidus stimulation timed and tailored provided symptomatic control in this cohort and may be considered in other carefully selected cases.

## Introduction

Adult-onset truncal dystonia (ATD) is more frequently reported in the context of severe segmental and generalized dystonic syndromes, and rarely as an isolated presentation of dystonia (Bhatia et al., 1997; Benecke and Dressler, 2007; Albanese et al., 2013; Lizarraga and Fasano, 2019). It accounts for about 10% of segmental dystonia and affects predominantly the trunk, including the paraspinal and abdominal wall muscles, with sparing or minimal involvement of the limbs and occasional contiguous spread to the cranio-cervical junction (Jabbari et al., 1992; Bhatia et al., 1997; Albanese et al., 2013; Shaikh et al., 2014). ATD is a major source of disability, occurring in either anteroflexion, retroflexion, lateroflexion or combined, and usually worsens with action or voluntary movement (Lizarraga and Fasano, 2019). A non-fixed forward bending of the trunk (>45 degrees) caused by hyperactivation of the rectus abdominis muscles is defined as camptocormia, which is the most common presentation of idiopathic ATD (Bhatia et al., 1997; Ehrlich and Frucht, 2016), although also described in association with Parkinson’s disease (Azher and Jankovic, 2005). Another form of ATD is the dystonic opisthotonus, which is characterized by a backward arching of the trunk and neck due to overactivation of the paraspinal extensor muscles (Bhatia et al., 1997; Benecke and Dressler, 2007; Ehrlich and Frucht, 2016). A less common presentation of ATD is the reversible lateral bending of the trunk, with a tendency to lean to one side, sometimes described as Pisa syndrome (Bhatia et al., 1997; Barone et al., 2016; Ehrlich and Frucht, 2016; Lizarraga and Fasano, 2019).

In terms of etiology, ATD is highly heterogeneous (Jabbari et al., 1992; Bhatia et al., 1997; Benecke and Dressler, 2007; Shaikh et al., 2014; Selikhova et al., 2015). It has been observed in genetic, idiopathic and acquired dystonic syndromes (Jabbari et al., 1992; Bhatia et al., 1997; Shaikh et al., 2014; Selikhova et al., 2015; Lizarraga and Fasano, 2019), and can have central and peripheral etiologies. It may be associated with parkinsonism and neuromuscular disorders (Stamelou et al., 2013; Lizarraga and Fasano, 2019) as well as with neuroleptic-induced acute and tardive dystonia (Bhatia et al., 1997; Benecke and Dressler, 2007; Barone et al., 2016).

Although botulinum toxin injections are generally the treatment of choice for adult-onset focal and segmental dystonia, the response of ATD to medical treatment, including botulinum toxin, is often limited due to the large number of muscles involved and consequently high total toxin dose required to produce the relevant clinical benefit (Bhatia et al., 1997; Benecke and Dressler, 2007; Zittel et al., 2009; Cloud et al., 2014; Shaikh et al., 2014; Mehta et al., 2020). Deep brain stimulation (DBS) in the treatment of generalized and segmental dystonia is now supported by robust evidence (Fox and Alterman, 2015). Nevertheless only few reports have specifically addressed the potential role of DBS in the management of dystonic opisthotonus in the context of truncal predominant adult-onset dystonia (Lizarraga and Fasano, 2019). Here we describe three cases of medically refractory ATD, where disabling dystonic opisthotonus was the main indication for bilateral globus pallidus internus (GPi) DBS.

## Materials and Methods

All three patients underwent comprehensive pre-operative multidisciplinary assessment prior to DBS intervention. Quantification of dystonia severity was performed using the Burke-Fahn-Marsden dystonia rating scale (BFMDRS; Burke et al., 1985). Quantification of overall pain severity was performed using the Visual Analog Scale (VAS). Quadripolar Medtronic 3389 DBS electrodes (Medtronic Inc., Minneapolis, MN, United States) were implanted in the GPi under general anesthesia as previously described (O’Gorman et al., 2011). The standard procedure consisted of preoperative stereotactic magnetic resonance imaging (MRI), using a 1.5 T General Electric (GE) MRI scanner (GE Healthcare, Chicago, IL, United States) or a 1.5 T Siemens MRI scanner (Siemens, Erlangen, Germany) on the day of surgery. MRI sequences for surgical planning and acquisition of the stereotactic coordinates consisted of volumetric T1-weighted and proton density-weighted scan for optimal visualization of the GPi, using a repetition time (TR) of 5,630 ms, an echo time (TE) of 15 ms, a slice thickness of 2 mm, field of view (FoV) of 250 mm, flip angle of 250 degrees, base resolution of 256 mm, and a voxel size of 0.5 mm × 0.5 mm × 2 mm. Stereotactic planning was based on the direct visualization of the targeted structure on the proton density sequence, where the posterior one-third of the ventral GPi was targeted for electrode placement. Microelectrode recording was not used in these cases. Surgery was performed in two stages, i.e., insertion of the intracranial leads (3389 electrodes, Medtronic Inc., United States) using the Leksell G frame (Elekta, Sweden), followed by placement of extensions and an implantable pulse generator (Medtronic Inc., United States) on the same day. Verification of the final position of the electrodes was performed with a postoperative high-definition computed tomography imaging of the head using an Optima 660 CT scanner (GE Healthcare, Chicago, IL, United States). Image processing and segmentation of the DBS leads were performed on LEAD-DBS software (Horn and Kühn, 2015; Edlow et al., 2019). Patients were assessed on a regular basis post-operatively in the multidisciplinary DBS clinic.

This study was approved by our institution’s Research Advisory Group and written informed consent was obtained from all patients. The study was carried out in accordance with The Code of Ethics of the World Medical Association (Declaration of Helsinki). All patients gave consent to be videoed for publication both in print and online.

## Results

**FIGURE 1:**
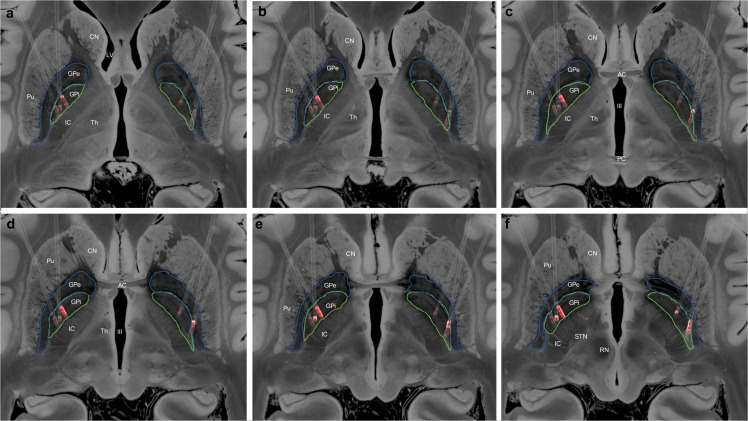
**(a–f)** Localization of the deep brain stimulation (DBS) electrodes, in standard space, for all patients. The Globus pallidus internus (GPi, green) and Globus pallidus externus (GPe, blue) have been manually segmented, and the active electrode contacts highlighted in red. CN, Caudate Nucleus; LV, Lateral Ventricle; Pu, Putamen; IC, Internal Capsule; Th, Thalamus; AC, Anterior Commissure; PC, Posterior Commissure; III, Third Ventricle; STN, Subthalamic Nucleus; and RN, Red Nucleus.

Three male patients with medically refractory ATD and severe dystonic opisthotonus, who had undergone GPi-DBS, were studied. The age at diagnosis ranged from 32 to 51 years and the mean duration of symptoms prior to surgery was 10.7 + 5.7 years (range 4–16 years). At the time of surgical treatment patients were 48 to 55 years old. The follow-up period ranged from 20 to 175 months. Table 1 summarizes the baseline characteristics, pharmacotherapy, stimulation settings, and outcomes. Figure 1 demonstrates the final position of the electrodes within the bilateral posteroventral GPi.

**TABLE 1:** Patient features, stimulation settings, and outcome.

	Case 1	Case 2	Case 3
Diagnosis	Neuroleptic induced tardive dyskinesia and dystonia	Adult-onset dystonia parkinsonism	Primary adult-onset axial dystonia
Age at diagnosis (years)/Gender	43/Male	51/Male	32/Male
Age at surgery (years)	55	55	48
Disease duration (years)	12	4	16
Medications pre-DBS (total daily dose)	Baclofen 70 mg, clonazepam 1,500 μg, olanzapine 7.5 mg	Baclofen 60 mg, procyclidine 15 mg, co-careldopa 625 mg, lorazepam 4 mg	Zopiclone 30 mg, tramadol 400 mg, benzhexol 2 mg, baclofen 20 mg, tetrabenazine 25 mg, oxazepam 120 mg
Medications post-DBS at last follow-up (total daily dose)	Baclofen 50 mg, clonazepam 1,000 μg, olanzapine 7.5 mg	Baclofen 60 mg, procyclidine 15 mg, co-careldopa 625 mg, Lorazepam 4 mg	Tramadol 400 mg, zopiclone 30 mg
Time to last follow-up (months)	144	20	175
Stereotactic coordinates (tip of the electrode verified postoperatively; mm)	AC-PC length = 24.34	AC-PC length = 24.70	AC-PC length = 27.41
	Left: *x* = −22.7; *y* = 3.1; *z* = −4.4	Left: *x* = −19.2; *y* = −0.4; *z* = −4.0	Left: *x* = −21.9; *y* = −0.2; *z* = −4.0
	Right: *x* = 21.9; *y* = 1.2; *z* = −2.8	Right: *x* = 22.2; *y* = −2.1; *z* = −3.0	Right: *x* = 20.3; *y* = −0.4; *z* = −3.0
Initial Stimulation settings	Left: C + 1-; 1.5 V, 60 ms, 130 Hz	Left: C + 2-; 0.5 V, 450 ms, 130 Hz	Left: C + 2-; 1.0 V, 450 ms, 130 Hz
	Right: C + 5-, 1.5 V, 60 ms, 130 Hz	Right: C + 10-, 0.5 V, 450 ms, 130 Hz	Right: C + 6-, 1.0 V, 450 ms, 130 Hz
Stimulation settings at 1-year follow-up	Left: C + 1-; 2.5 V, 60 ms, 130 Hz	Left: C + 1–2-; 0.5 V, 360 ms, 130 Hz	Left: C + 1–2-; 2.0 V, 450 ms, 140 Hz
	Right: C + 5-, 2.5 V, 60 ms, 130 Hz	Right: C + 10– 9-, 0.5 V, 360 ms, 130 Hz	Right: C + 5– 6-, 2.0 V, 450 ms, 140 Hz
Stimulation settings at last follow-up	Left: C + 1-; 3.5 V, 90 ms, 130 Hz	Left: C + 1–2-; 0.5 V, 360 ms, 130 Hz	Left: C + 0–1–2-; 1.8 V, 450 ms, 140 Hz
	Right: C + 5-, 3.5 V, 90 ms, 130 Hz	Right: C + 5-, 0.5 V, 360 ms, 130 Hz	Right: C + 4–5– 6-, 1.7 V, 450 ms, 140 Hz
	IPG Kinetra (eventually replaced by Activa PC)	IPG Activa PC	IPG Kinetra (eventually replaced by Activa PC)
Preoperative BFMDRS-M	Preoperative: 34	Preoperative: 26	Preoperative: 36
Postoperative BFMDRS-M (1-yr FU)	Postoperative: 9.5	Postoperative: 7.5	Postoperative: 10
% improvement BFMDRS-M (1-yr FU)	Improvement: 72	Improvement: 71.2	Improvement: 72.2
Postoperative BFMDRS-M (last FU)	Postoperative: 7.5	Postoperative 7.5	Postoperative: 4
% improvement BFMDRS-M (last FU)	Improvement: 77.9	Improvement 71.2	Improvement: 88.9
Preoperative BFMDRS-D	Preoperative: 9	Preoperative: 5	Preoperative: 13
Postoperative BFMDRS-D (1-yr FU)	Postoperative: 4	Postoperative: 2	Postoperative: 5
% improvement BFMDRS-D (1-yr FU)	Improvement: 55.6	Improvement: 60	Improvement: 61.5
Postoperative BFMDRS-D (last FU)	Postoperative: 4	Postoperative: 2	Postoperative: 4
% improvement BFMDRS-D (last FU)	Improvement: 55.6	Improvement: 60	Improvement: 69.2
Preoperative Subscores for neck	Preoperative: 8	Preoperative: 8	Preoperative: 8
Postop. subscores for neck (1-yr FU)	Postoperative: 2	Postoperative: 2	Postoperative: 1
% improvement subscore (1-yr FU)	Improvement: 75	Improvement: 75	Improvement: 87.5
Postop. subscores for neck (last FU)	Postoperative: 0	Postoperative: 2	Postoperative: 1
% improvement subscore (last FU)	Improvement: 100	Improvement: 75	Improvement: 87.5
Preoperative Subscores for trunk	Preoperative: 16	Preoperative: 12	Preoperative: 16
Postop. subscores for trunk (1-yr FU)	Postoperative: 2	Postoperative: 3	Postoperative: 6
% improvement subscore (1-yr FU)	Improvement: 87.5	Improvement: 75	Improvement: 62.5
Postop. subscores for trunk (last FU)	Postoperative: 0	Postoperative: 3	Postoperative: 1
% improvement subscore (last FU)	Improvement: 100	Improvement: 75	Improvement: 93.7
Preoperative VAS	Preoperative: 8/10	Preoperative: 8/10	Preoperative: 8/10
Postoperative VAS	Postoperative: 0/10	Postoperative: 4/10	Postoperative: 3/10
% improvement VAS (last FU)	Improvement: 100	Improvement: 50	Improvement: 62.5

*DBS, deep brain stimulation; BFMDRS-M, motor component of Burke-Fahn-Marsden Dystonia Rating scale; BFMDRS-D, disability component of Burke-Fahn-Marsden Dystonia Rating Scale; VAS, visual analog scale for pain; 1-yr FU, outcome at 1-year follow-up; and last-FU, outcome at last follow-up appointment.*

### Case 1

This 67-year-old man was diagnosed with severe tardive dystonia at the age of 43 years, following 9 years of neuroleptic treatment for schizophrenia. His psychiatric disorder had been fully controlled for many years. Over the subsequent 12 years, blepharospasm, facial grimacing and limb dyskinesia were mild, but he had much more severe and disabling dystonic opisthotonus. He could only mobilize with a *geste antagoniste* by voluntarily flexing his trunk forward to 90 degrees. Neuroradiological and biochemical work-up were unremarkable. Preoperative BFMDRS-M score was 34.

Attempts to treat him with clozapine and quetiapine were unhelpful due to sleepiness. Low dose Olanzapine could not be withdrawn as this led to persecutory ideas returning. After further follow-up by both neurologists and psychiatrists, and no evidence of active psychiatric symptoms, bilateral GPi DBS was carefully discussed in our MDT. The patient was 55 years old when surgery was performed. Dramatic improvements of his symptoms were noted upon electrical stimulation starting from the next day (Supplementary Video 1, segments 1 and 2). Over the next few months, his quality of life improved to the extent that he was able to participate in social activities, including for the first time in many years going to restaurants, and to spectate at family, school and sports fixtures. During the first 2 years the stimulations settings had to be adjusted at times, until stable and satisfactory response was finally achieved. The stimulation settings have been stable for the last 7 years. At the last follow up appointment (144 months), he had minimal evidence of retrocollis or abnormal truncal movements. Improvements of 77.9% in BFMDRS-M and 100% in VAS scores were noted. Detailed pre- and postoperative outcome scores, including BFMDRS subscores for trunk and neck are presented in Table 1.

### Case 2

This 56-year-old man developed progressive trunk hyperextension, with backward spasms and lateral flexion over a period of 6 months. The onset of his symptoms was at the age of 51 years, without any prior medical history. He also had intermittent chin tuck while sitting and, mild tongue protrusion and involuntary backwards jerking of his neck. Truncal retroflexion was significantly aggravated on walking, and only modestly relieved by *geste antagoniste*, such as by touching the back of his head or leaning against the wall. Extensive investigations for structural and inflammatory causes did not confirm a diagnosis. Botulinum toxin to the posterior neck muscles was helpful for neck spasms, but the arching back was felt to be too extensive to treat with botulinum injections.

Combinations of medical treatment were unhelpful. A short trial of low dose olanzapine was successful in suppressing his truncal retroflexion, but he quickly developed new parkinsonian side effects of limb bradykinesia, rigidity and jaw tremor. Stopping olanzapine resolved parkinsonism but at the expense of return of original truncal retroflexion. A DaT-scan showed bilateral nigrostriatal dysfunction, which we did not feel was attributable to his medications, nor was this a typical initial presentation of parkinsonism – dystonia.

Genetic tests including for ATP1A3 (DYT12), PANK2, PLA2G6, Wilson’s and a further search for the possibility of hitherto undisclosed intake of dopaminergic antagonists were not helpful. No Philippines ancestry was noted. On the rare possibility that the DaT-scan was an erroneous false positive, it was repeated and again showed bilaterally reduced uptake in the striatum. Consideration to adult-onset dopamine transporter deficiency syndrome was given, despite his age. CSF analysis for HVA/5HIAA and neurotransmitters was normal. A minor abnormality in CSF folate metabolism was discounted as relevant because a trial period of Folinic acid supplementation was unhelpful. SLC6A3 and whole genome sequencing were negative. Muscle biopsy was negative for mitochondrial disease. Levodopa had little benefit and a trial of dopamine agonist significantly exacerbated the dystonia. We cannot formally diagnose this disorder, so it is presently pragmatically labeled as adult-onset dystonia-parkinsonism. Preoperative BFMDRS-M score was 26.

Bilateral GPi DBS was carried out at the age of 55 years. The patient presented a remarkable improvement of his symptoms on the first postoperative day, likely due to microlesioning effect, which lasted around 3 weeks (Supplementary Video 1, segments 3 and 4). In the monopolar review he presented marked bilateral akinesia or worsening of the truncal dystonia with higher stimulation amplitudes. Nevertheless, further adjustments of the stimulation settings during the following 12 months led to complete resolution of the retrocollis and about 65% improvement of his truncal hyperextension. At last postoperative follow-up (20 months), the dystonic opisthotonus and lateral bending were satisfactorily controlled, allowing independent mobility with near abolition of the involuntary neck and back jerks. An improvement of 71.2% in BFMDRS-M and 50% in VAS scores were noted. Detailed BFMDRS subscores for neck and trunk are presented in Table 1.

### Case 3

This 62-year-old man was diagnosed with idiopathic adult-onset axial dystonia at the age of 32 years. Initial symptoms were blepharospasm, minor swallowing symptoms and torticollis, which responded to Botulinum toxin injections. Within 16 years, his dystonia progressed to profound axial trunk dystonia with worsening balance, falling backwards and requiring a wheelchair to mobilize because of the severe dystonic opisthotonus. When standing, he could only walk with his trunk flexed and hands on his knees, and he transferred by crawling. Multiple investigations for etiologies were negative and medical therapies were largely ineffective for his truncal retroflexion. Levodopa worsened his symptoms. Genetic testing did not identify a diagnosis. Preoperative BFMDRS-M score was 36.

Bilateral GPi DBS was implanted at 48 years of age. Immediate blepharospasm improvement and gradual resolution of back jerks were noted with therapy initiation. Over the next few months, axial dystonia control was achieved, allowing him to sit, walk straighter and for longer distances (Supplementary Video 1, segments 5 and 6). He was able to perform daily activities as well as return to driving. Following surgery, the optimization phase of DBS settings took around 12 months to reach stable settings and good control of dystonic symptoms. However, following a DBS battery change at 7 years follow-up, he presented with worsening of the retrocollis, which resolved after further adjustments of the stimulation parameters. Investigation with neuroimaging did not reveal any sings of electrode displacement. The DBS settings have been stable since then. At last follow-up (175 months), his dystonic symptoms were well controlled with an 88.9% improvement in the BFMDRS-M and 62.5% in the VAS scores.

## Discussion

Dystonia is one of the most disabling movement disorders, having a significant impact on the patient’s quality of life (Fox and Alterman, 2015; Rodrigues et al., 2019). Rehabilitation and medical management along with local botulinum toxin injections are the treatment of choice in focal dystonia (Albanese and Lalli, 2012; Ehrlich and Frucht, 2016). Since segmental and generalized forms of dystonia may not respond well to pharmacological therapies, there has been growing interest and expansion in the application of bilateral neuromodulation of deep brain structures in the management of these challenging conditions (Zittel et al., 2009; Martínez et al., 2014; Shaikh et al., 2014; Fox and Alterman, 2015; Tambirajoo et al., 2020; Furlanetti et al., 2021). ATD is more frequently reported in the context of severe segmental and generalized dystonic syndromes, and rarely as an isolated presentation of dystonia (Bhatia et al., 1997; Benecke and Dressler, 2007; Albanese et al., 2013; Lizarraga and Fasano, 2019).

Adult-onset dystonic opisthotonus as a main feature is rare and has been described as a “red-flag” sign for drug-induced dystonia, neurometabolic disorders (Wilson Disease, Lesch-Nyhan Syndrome, Maple syrup urine disease) and neurodegeneration with brain iron accumulation (NBIA; Stamelou et al., 2013). The clinical management of ATD, including forms presenting predominantly with trunk and neck hyperextension is challenging as it often fails to respond to toxin injection (Benecke and Dressler, 2007). Bhatia el al reported a historical cohort of 18 patients with axial adult-onset primary dystonia, where 55% of patients had trunk anteroflexion, 22% trunk hyperextension, and 5.6% lateral bending. The overall response to medical treatment was poor, where only 16.6% had moderate and 22% had pronounced improvement (Bhatia et al., 1997). In line with this, Comella et al. reported their experience in the management of dystonic opisthotonus with botulinum toxin in 5 patients. The mean overall improvement in truncal dystonia subscore was 37%, which was also associated with reduction of pain (Comella et al., 1998).

Deep brain stimulation is now well established as an effective treatment in primary generalized, segmental and cervical dystonia (Eltahawy et al., 2004; Andrews et al., 2010; Koy et al., 2013; Vidailhet et al., 2013; Fox and Alterman, 2015; Moro et al., 2017; Lizarraga and Fasano, 2019; Rodrigues et al., 2019). There is, however, little literature on its effectiveness on medically refractory ATD (Nandi et al., 2002; Sakas et al., 2010; Capelle et al., 2011; Shaikh et al., 2014; Lizarraga and Fasano, 2019; Mohd Fauzi et al., 2019; Rodrigues et al., 2019).

Our clinical study aimed to evaluate the role of bilateral GPi-DBS in the management of adult-onset dystonic opisthotonus in the context of trunk predominant dystonia. Although our patients presented with generalized dystonic symptoms, affecting not only the trunk but to a minor extent also the cranio-cervical and brachial regions, the dystonic opisthotonus was the main disabling problem, and therefore the indication for surgical treatment. The exemplary clinical cases presented cover a range of etiologies, which demanded varying degrees of investigation. The first patient had clear diagnosis of a tardive dystonic syndrome, whereas the etiologies for the remaining two cases could not be determined, despite of extensive neuroradiological and genetic testing. Additionally, we describe the impact of severe dystonic symptoms on each individual patient, including social functioning, as well as difficulties encountered in overall clinical and surgical managements.

Tardive dystonia is a complication of the chronic exposure to dopamine receptor blocking agents (Truong and Frei, 2019). The more common stereotypical movements occur in 15–20% of patients on neuroleptics and dystonic movements occur in 1–4% (Raja, 1995; Adityanjee et al., 1999). The remission rate is less than 15% and occurs on average 2.6 years after discontinuation of the causative agent (Cloud et al., 2014). It is associated with a high incidence of morbidity and mortality (Youssef and Waddington, 1987). GPi DBS has been reported to improve tardive dystonia by more than 90% (Trottenberg et al., 2005; Cohen et al., 2007; Gruber et al., 2009; Shaikh et al., 2015). Our case adds to previous evidence, emphasizing the need for meticulous neurological and psychiatric evaluation before, during and after DBS.

Dystonia is also reported to occur in 30% of patients with Parkinson’s disease (PD; Kidron and Melamed, 1987). It is commonly observed in young onset PD and autosomal recessive genetic parkinsonism (LeWitt et al., 1986; Shetty et al., 2019). The underlying pathophysiological mechanism is poorly understood. The dystonia may be a presenting symptom of PD and can precede the typical clinical symptoms of PD by up to a decade (Tolosa and Compta, 2006). Response to dopamine replacement therapy in early dystonia is variable (LeWitt et al., 1986). Additionally, levodopa therapy in itself can cause dystonia (Monville et al., 2009; Shetty et al., 2019). GPi DBS is usually an effective treatment for both dystonia and parkinsonism in Parkinson’s patients (Lizarraga and Fasano, 2019; Shetty et al., 2019). Despite extensive investigations, the diagnosis in case 2 described here remains uncertain. The abnormal DaT-scan and certain clinical features emerging later suggest parkinsonism but to the best of our knowledge, dystonic opisthotonus as an isolated initial symptom of PD is rare (Lizarraga and Fasano, 2019).

Despite of initial poor response to medical management, toxin injection and rehabilitation therapies, all patients showed rapid and long-lasting responses to bilateral GPi-DBS. The overall percentage improvement in the BFMRDS at 1-year ranged from 63.8 to 66.8%, improving to 66.7–79% in the long-term follow-up (Table 1). Furthermore, all patients had considerable improvement with respect to dystonic pain at the long-term follow-up (VAS% improvement, range 50–100%, Table 1). Given that the overall BFMDRS may not accurately reflect the impact of the GPi-DBS on the axial symptoms, we further analyzed the BFMDRS sub-scores for neck and trunk, showing an even greater impact of DBS at the last follow-up, with 75–100% improvement.

These findings are in line with previous reports of ATD successfully treated with DBS (Nandi et al., 2002; Sakas et al., 2010; Capelle et al., 2011; Shaikh et al., 2014; Lizarraga and Fasano, 2019; Mohd Fauzi et al., 2019). In a single case report of a patient with flexion and lateral flexion subtypes, Zittel et al. (2009) showed alleviation of the axial dystonia by GPi DBS. In another series of 4 patients with both flexion and extension subtypes, BFMDRS scores improved by 30% in the first month and over 80% at 2 years (Shaikh et al., 2014). The authors noted that a higher voltage and longer pulse width correlated with better outcomes (Zittel et al., 2009; Shaikh et al., 2014). In their systemic review, Lizarraga et al. recently highlighted varied response rates of trunk postural deformities to DBS. Thus, improvement was noted as 59% in Parkinsonian camptocormia, 50–100% in dystonic camptocormia and 33–66.7% in Parkinsonian Pisa syndrome (Lizarraga and Fasano, 2019). Interestingly, only 2 cases of truncal and neck hyperextension were identified, both in patients with onset of dystonia during childhood, underpinning the rarity of ATD with predominant dystonic opisthotonus (Sakas et al., 2010; Mohd Fauzi et al., 2019). Mohd Fauzi et al. (2019) described a 25-year-old patient with generalized dystonia and predominantly severe neck and trunk hyperextension associated with NBIA, who underwent bilateral GPi-DBS, obtaining a rapid response and 83.3% improvement in BFMDRS trunk subscores, at 2-year-follow-up. Sakas et al. (2010) reported a 29-year-old male with previous history of perinatal hypoxia, confined to bed since the age of 9 years, due to severe dystonic opisthotonus. The patient underwent bilateral GPi-DBS with 61.5% overall improvement in BFMDRS. Due to his long-standing severe dystonic syndrome and some degree of skeletal deformities, the fixed component of dystonia did not improve completely after DBS, as also previously reported (Jankovic, 2008; Lozano et al., 2009; Albanese et al., 2013). Nevertheless, GPi neuromodulation allowed the patient to walk again and climb stairs unaided (Sakas et al., 2010). This was consistent with the findings in 2 of our patients (cases 1 and 3), who had extraordinary improvement of the mobile component of their axial dystonia following GPi-DBS but did remain with a degree of fixed truncal deformity (Supplementary Video 1).

Overall, our series shows that the patients garnered significant control of their dystonic symptoms, with remarkable improvement of the dystonic opisthotonos following DBS, allowing them to reintegrate into their personalized environment and society, aiming toward a normal life. Dystonic symptoms re-emerged rapidly when DBS battery were near or completely depleted. Benefits were reinstated upon battery revision and were maintained in the long-term with no associated morbidity.

## Conclusion

Adult-onset dystonic syndromes predominantly presenting with dystonic opisthotonus are relatively rare. The specific nature of dystonic opisthotonus remains a treatment challenge, and thorough investigation of this highly disabling condition with varying etiologies is often necessary. Although patients may be refractory to medical management and botulinum toxin injection, Globus pallidus stimulation timed and tailored provided symptomatic control in this cohort and may be considered in other carefully selected cases.

## Data Availability Statement

The original contributions presented in the study are included in the article/Supplementary Material, further inquiries can be directed to the corresponding author/s.

## Ethics Statement

The studies involving human participants were reviewed and approved by King’s College Hospital NHS Foundation Trust Research Advisory Group. The patients/participants provided their written informed consent to participate in this study. Written informed consent was obtained from the individual(s) for the publication of any potentially identifiable images or data included in this article.

## Author Contributions

KT and LF organized, executed, wrote, and reviewed the study. MS and KA conceived, organized, supported and reviewed the study, and contributed equally as senior authors. All authors have seen and approved the final version of manuscript being submitted.

## Conflict of Interest

KA has received education grants and honoraria from Medtronic, Abbott Medical and Boston Scientific companies. MS has received educational grant from Medtronic and Abbott Medical and acts as a consultant for Abbott Medical. King’s College Hospital received Education support for educational meetings from Parkinson’s United Kingdom. The remaining authors declare that the research was conducted in the absence of any commercial or financial relationships that could be construed as a potential conflict of interest.

## Supplementary Material

Supplementary Video 1 *Case 1*: preoperative examination standing/walking (segment 1); first postoperative day (segment 2); *Case 2*: preoperative neurological examination in sitting position and standing/walking (segment 3); first postoperative day (segment 4); and *Case 3:* preoperative examination standing/walking (segment 5); postoperative evaluation (segment 6).
